# Evaluating networked drug checking services in Toronto, Ontario: study protocol and rationale

**DOI:** 10.1186/s12954-019-0336-0

**Published:** 2020-01-28

**Authors:** N. Maghsoudi, K. McDonald, C. Stefan, D. R. Beriault, K. Mason, L. Barnaby, J. Altenberg, R. D. MacDonald, J. Caldwell, R. Nisenbaum, P. Leece, T. M. Watson, K. W. Tupper, L. Kufner, A. I. Scheim, D. Werb, Drug Checking Services in Toronto Working Group

**Affiliations:** 1 Centre on Drug Policy Evaluation, Li Ka Shing Knowledge Institute, St. Michael’s Hospital, 30 Bond Street Toronto, Ontario, M5B 1 W8 Canada; 2 Institute of Health Policy, Management and Evaluation, University of Toronto, Toronto, Ontario Canada; 3 Clinical Laboratory and Diagnostic Services, Centre for Addiction and Mental Health, Toronto, Ontario Canada; 4 Department of Laboratory Medicine, St. Michael’s Hospital, Toronto, Ontario Canada; 5 Department of Laboratory Medicine and Pathobiology, University of Toronto, Toronto, Ontario Canada; 6 South Riverdale Community Health Centre, Toronto, Ontario Canada; 7 Parkdale Queen West Community Health Centre, Toronto, Ontario Canada; 8 Toronto Paramedic Services, Toronto, Ontario Canada; 9 Faculty of Medicine, University of Toronto, Toronto, Ontario Canada; 10 Toronto Public Health, Toronto, Ontario Canada; 11 MAP Centre for Urban Health Solutions and Applied Health Research Centre, Li Ka Shing Knowledge Institute, St. Michael’s Hospital, Toronto, Ontario Canada; 12 Dalla Lana School of Public Health, University of Toronto, Toronto, Ontario Canada; 13 Public Health Ontario, Toronto, Ontario Canada; 14 Department of Family and Community Medicine, University of Toronto, Toronto, Ontario Canada; 15 Institute for Mental Health Policy Research, Centre for Addiction and Mental Health, Toronto, Ontario Canada; 16 School of Population and Public Health, University of British Columbia, Vancouver, British Columbia Canada; 17 Division of Infectious Diseases and Global Public Health, University of California San Diego School of Medicine, La Jolla, CA USA

**Keywords:** Drug checking services, Harm reduction, Overdose, Drug market monitoring

## Abstract

**Background:**

The increasing incidence of fatal opioid overdose is a public health crisis in Canada. Given growing consensus that this crisis is related to the presence of highly potent opioid adulterants (e.g., fentanyl) in the unregulated drug supply, drug checking services (DCS) have emerged as part of a comprehensive approach to overdose prevention. In Canada’s largest city, Toronto, a network of DCS launched in 2019 to prevent overdose and overdose-related risk behaviors. This network employs mass spectrometry technologies, with intake sites co-located with supervised consumption services (SCS) at three frontline harm reduction agencies. The protocol and rationale for assessing the impact of this multi-site DCS network in Toronto is described herein. The aims of this study are to (1) evaluate the impact of DCS access on changes in and factors influencing overdose and related risk behaviors, (2) investigate the perceived capacity of DCS to prevent overdose, and (3) identify composition (qualitative and quantitative) trends in Toronto’s unregulated drug supply.

**Methods:**

We will use a parallel-mixed-methods design with complementary data sources (including data from chemical analysis of drug samples, quantitative intake and post-test surveys, SCS, coroners, paramedic services, and qualitative interviews), followed by a meta-inference process wherein results from analyses are synthesized.

**Results:**

Whereas most DCS globally target “recreational drug users,” in Toronto, this networked DCS will primarily target marginalized people who use drugs accessing frontline services, many of whom use drugs regularly and by injection. This evolution in the application of DCS poses important questions that have not yet been explored, including optimal service delivery models and technologies, as well as unique barriers for this population. Increasing information on the unregulated drug supply may modify the risk environment for this population of people who use drugs.

**Conclusions:**

This study addresses evidence gaps on the emerging continuum of overdose prevention responses and will generate critical evidence on a novel approach to reducing the ongoing high incidence of drug-related morbidity and mortality in Canada and elsewhere.

## Background

The increasing incidence of fatal opioid overdose is a public health crisis in Canada. From January to September 2018, there were 3286 opioid-related deaths in Canada, corresponding to a death rate of 11.8 per 100,000 population (compared to 11.1 and 8.4 per 100,000 population in 2017 and 2016, respectively) [1]. In the western province of British Columbia, opioid overdose deaths have contributed to a decline in life expectancy, particularly among the most socioeconomically disadvantaged and marginalized communities [2]. In the province of Ontario, opioid overdose is now the leading cause of accidental death for young people, with one of every eight deaths among individuals aged 25 to 34 years involving an opioid [3]. With 308 opioid-related deaths in 2017, the country’s largest city, Toronto, Ontario, has experienced a 125% increase in deaths compared to 2015 [4]. There is now a consensus that the rising incidence of overdose is related in large part to the emergence of highly potent opioids, such as fentanyl and fentanyl analogs, in the unregulated drug supply. For example, fentanyl is present in an increasing number of overdose fatalities in Canada, though overdose deaths excluding fentanyl have remained relatively stable [5]. In Ontario, the presence of fentanyl in opioid-related deaths increased by 548% between 2006 and 2015 [6]. In 2017, there were 1265 opioid-related deaths in the province and fentanyl was present in 63.6% of these deaths; in contrast, fentanyl was present at death in 40.7% of 867 opioid-related deaths in 2016 [7]. This trend has thus accelerated in recent years, and fentanyl has overtaken heroin as the opioid most commonly present in opioid overdose deaths in Toronto [4]. In response, Canada’s federal government has characterized the current situation as a national public health crisis and allocated CAD$116 million in 2017 to a public health emergency response coordinated by Health Canada [8, 9], while the Government of Ontario committed CAD$280 million over 3 years to address the opioid overdose crisis [10].

Against this backdrop [11], drug checking services (DCS) have emerged as part of a comprehensive approach to preventing overdose mortality [12, 13]. Stakeholders—including the Federal Standing Committee on Health [14], Toronto Board of Health [15], and Mayors’ Task Force on the Opioid Crisis [16]—have identified DCS as a critical tool within a comprehensive plan to prevent overdoses related to the use of high-potency unregulated opioids. As a harm reduction intervention available in Europe since the 1990s, DCS provide information on the composition of drugs to their clients in order to facilitate more informed drug-related decision-making and to increase the capacity of individuals to avoid ingesting unanticipated toxic substances, which can lead to overdose and death [17, 18]. By aggregating analytic data on drug samples, DCS can also provide insight into trends in the unregulated drug supply [19–26]. The Canadian federal government has identified DCS as a key component of their response to the opioid overdose crisis and has committed to authorizing and funding pilot projects providing DCS at supervised consumption services (SCS) [27]. This includes funding over 5 years for the implementation of a multi-site DCS network in Toronto with the primary aim of reducing overdose deaths among structurally vulnerable individuals reliant on the unregulated opioid supply [28]. Launched in 2019, the DCS network in Toronto is located within frontline harm reduction agencies offering SCS and employs liquid chromatography and gas chromatography-mass spectrometry (LC- and GC-MS), the gold standards in forensic drug analysis [29], making Toronto’s DCS unique among Canadian DCS [30].

To date, evaluations of DCS have been limited to “recreational drug users” in nightlife settings in Europe [19, 31, 32], and no DCS employing LC- or GC-MS analysis for point of care services and operating within SCS have been scientifically evaluated. Herein, we describe the methodology for a study assessing the impact of a multi-site DCS network in Toronto. The overall goal of the this study is to determine whether access to DCS, to be co-located with SCS at three frontline harm reduction agencies in Toronto, is associated with a reduction in the incidence of overdose and related risk behaviors resulting from the consumption of unknown or toxic unregulated drugs by people who use drugs accessing frontline services, including young people who use drugs. The study has three specific aims: (1) evaluate the impact of DCS access on changes in and factors influencing overdose and related risk behaviors, (2) investigate the perceived capacity of DCS to prevent overdose, and (3) identify composition (qualitative and quantitative) trends in Toronto’s unregulated drug supply. Given the limited evidence on the influence and impact of DCS for subpopulations of people who use drugs regularly or by injection, this research study will represent a novel contribution to the scientific literature and provide important insights on DCS as an overdose prevention intervention. The protocol and rationale for this study is itself an important contribution to the literature, as it provides full study transparency and will be useful to researchers planning to evaluate DCS in other jurisdictions.

## Methods

### Conceptual framework

We employed Rhodes’ risk environment framework, which posits that physical, social, economic, and policy environments influence an individual’s health decision-making [33]. Drug policy changes in Canada—such as expanded access to naloxone (a medication that blocks the effects of opioid overdose), the adoption of legislation providing amnesty from drug possession charges for those calling emergency services in an overdose situation, and the scale-up of SCS—have modified the risk environment for people who use drugs to allow this population to avoid a range of drug-related risks. However, these responses are generally limited to addressing the needs of individuals during or after drug use, rather than before. In contrast, DCS are intended to provide people who use drugs with critical information so they can make informed decisions and take action prior to their use of unregulated drugs, thereby possibly preventing overdose from occurring in the first place. This is important given that an examination of the current risk environment experienced by people who use drugs in Toronto suggests that, while the emergence of fentanyl and fentanyl analogs in the unregulated drug supply is a macro-level factor in the increased incidence of fatal overdose and drug-related harm, social (e.g., experiences of trauma, ongoing stigma), economic (e.g., poverty), and policy (e.g., criminalization of drugs, lack of information on drug supply, low levels of access to social services) barriers constrain the capacity of individuals to avoid overdose risk [34, 35]. Applying Rhodes’ risk environment framework to this research study, DCS are defined as meso-level interventions that have the capacity to expand the range of choices that individuals reliant on the unregulated drug supply have to avoid overdose. This conceptual framework will guide variable selection for quantitative analyses and domains of interest for qualitative interviews. For instance, the study will examine the neighborhood in which drugs were purchased given that this factor shapes the meso- and micro-level economic risk environments [36] experienced by participants that may influence overdose risk. Gender and age will also be considered in the study to situate experiences of access to DCS and resulting outcomes given that the risk environment for drug-related harms is different across genders and ages [37, 38]. Qualitative interviews will investigate the influence of DCS on individuals’ perceived barriers to adopting overdose risk reducing behaviors.

### Approach

By employing complementary data sources—including data from analysis of drug samples, quantitative intake and post-test surveys, SCS, coroners, paramedic services, and qualitative interviews—this study will comprehensively evaluate the impact of DCS on a range of policy-relevant drug-related outcomes, while considering the impacts of co-located SCS (refer to Table 1: Measures, Outcomes, and Data Sources). A mixed-methods approach followed by a meta-inference process will be used to maximize multiple data sources and synthesize results of quantitative and qualitative analyses.

**Table 1:** Measures, outcomes, and data sources

Measures and outcomes	Data sources
Aim 1: Evaluate the impact of DCS access on changes in and factors influencing overdose and related risk behaviors	
Self-reported overdose among those that access DCS and those that do not	– Quantitative survey data (intake) – SCS client data
Proportion of participants reporting increase in protective behaviors (not using alone, carrying naloxone, use of SCS, consultation with staff, smaller/) “tester” dosage, discarding toxic substances)	– Quantitative survey data (intake/post-test) – SCS client data
Proportion of participants reporting they gained, intend to use, and/or used knowledge and skills	– Quantitative survey data (post-test) – Qualitative interviews
Aim 2: Investigate the perceived capacity of DCS to prevent overdose	
Characterize participant perceptions on the capacity of DCS to alter the risk of overdose	– Qualitative interviews
Identify participant perceptions of contexts, facilitators, and barriers to the use of DCS	– Qualitative interviews
Aim 3: Identify trends in the composition (qualitative and quantitative) of the unregulated drug supply in Toronto	
Number of analysis results detecting composition different from participant expectations	– Quantitative survey data (intake) – Drug sample analysis data
Increase in accuracy and timeliness of alerts in response to dangerous drug trends	– Drug sample analysis data – Qualitative interviews
Spatial association between frontline harm reduction agencies with DCS and fatal overdose, and changes over time	– First responder and coroner data (from OCC and TPaS)

### Study partners

Three frontline harm reduction agency partners—Parkdale Queen West Community Health Centre (Queen West site), South Riverdale Community Health Centre, and The Works at Toronto Public Health—have been funded as DCS intake sites. With the exception of The Works, which is a public health agency, the remaining two partnering frontline agencies are community health centers, and all agencies combine harm reduction service delivery with access to other services, including primary care, mental health care, and social service programs. As DCS intake sites, agencies will collect drug samples from participants, administer intake and post-test surveys, facilitate transport of samples to clinical laboratories, and communicate analysis results to participants along with relevant harm reduction information. Notably, Parkdale Queen West Community Health Centre (Queen West site) also houses TRIP Project, a program providing information and resources on harm reduction and safer sex to youth in nightlife and party settings.

Clinical laboratory partners at the Centre for Addiction and Mental Health and St. Michael's Hospital—two urban hospitals with specialized programs for substance use—will analyze samples of drugs from participants at frontline harm reduction agencies using LC- and GC-MS techniques and guide the aggregation of analysis data across sites to identify composition (qualitative and quantitative) trends in the unregulated drug supply.

To bolster the relevance of findings and ensure effective knowledge translation strategies that address the needs of key populations, a Community Advisory Board made up of people who use drugs will advise on all aspects of the design, analysis, interpretation, and knowledge translation activities of this study [39]. Additionally, protocols for research methods and outcome measures will be aligned as much as possible with DCS implemented in other Canadian jurisdictions, including British Columbia and the capital city of Ottawa, Ontario, to maximize the potential comparability of findings across distinct settings.

### Data collection

#### Intake and post-test surveys (Aim 1)

Intake and post-test surveys will be administered to all consenting DCS clients at the three sites in Toronto, including participants who access DCS once and those who access DCS multiple times, allowing for a combined use of cross-sectional and longitudinal approaches. Surveys will be administered by frontline staff on an electronic tablet with the option for self-administration depending on the needs of the participant. Guided by the abovementioned conceptual framework, demographic and drug-using factors will be collected at first visit to DCS via a baseline intake survey to assess the influence of additional vulnerabilities facing key populations. An anonymous identifier will be created in the baseline intake survey and linked to intake surveys completed at subsequent visits. Questions on the intake survey, administered at every visit to DCS, include the following: What substance participants believe they are having tested?; When and where they purchased the substance?; and, If they are sharing the results with others, with how many people? Post-test surveys conducted immediately after analysis results are provided will query participants about their intended behaviors in response to analysis results, as well as allow frontline staff to record other services accessed (e.g., SCS, naloxone) and observed behaviors (e.g., discarding remainder of drug that was tested).

#### Sample size (Aim 1)

In lieu of randomization, which is neither feasible nor ethical given the type of service to be examined in the present study, a community recruitment model will be employed whereby a survey of all consenting DCS clients is undertaken (i.e., the sampling frame consists of all clients of DCS). Since study participants are recruited “passively” through their use of the service, we are unable to estimate the proportion of surveys to be obtained from unique versus repeat clients until after DCS are implemented. We therefore cannot determine the total person-time data that will be collected. We note, however, that 75% of feasibility survey respondents recruited from frontline agencies reported being willing to access DCS. The total population of structurally vulnerable people who use drugs in Toronto is estimated at 9000, among whom 20% are willing to use SCS [35]; it is estimated that *n* = 1800 unique individuals who use drugs access frontline agencies. Previous literature suggests social desirability bias in reports of willingness to access harm reduction services [40]; we therefore estimate an approximate total of *n* = 1080 DCS clients.

#### SCS client survey data (Aim 1 substudy)

An ongoing longitudinal cohort is collecting data from people who inject drugs who do and do not use SCS at the three frontline agencies participating in the DCS network. This allows for harmonization of survey tools across both projects. As such, for participants consenting to linkages, data from a standardized SCS cohort survey (e.g., age, gender, neighborhood of residence, and drug-using factors) and data from the SCS pre/post injection assessment form (e.g., overdose outcomes, whether emergency medical attention was provided [i.e., SCS staff response and/or call to 911 for paramedic response], naloxone administered) will be accessible for consenting participants. This will also allow for a substudy consisting of a comparison of outcomes (i.e., overdose incidence and related risk behaviors) among frontline agency clients who access SCS exclusively, who access both SCS and DCS, and who access neither.

#### Qualitative interviews (Aim 2)

In-depth interviewing will investigate participant perceptions of the contexts, facilitators, and barriers to the use of DCS and its capacity to influence overdose risk. We will also specifically investigate participant perspectives on the influence of gender on both DCS access and overdose risk, given the lack of data on this topic and the known contribution of gender to drug-related harms (i.e., women are less likely to manage illegal drug acquisition and inject themselves compared with men) [41–44]. We will employ purposive sampling to achieve gender diversity (including transgender individuals) and to ensure youth representation drawn from Toronto’s structurally vulnerable drug-using population [35]. Two interview waves will be conducted, and both will include 10 interviews with participants from each of the three SCS/DCS sites, which includes 5 interviews with youth accessing DCS via the TRIP Project (total *n* per wave = 30; total *n* = 60). Two waves of interviews will also be held with institutional stakeholders providing DCS services (*n* = 10 per wave; *n* = 20) to provide insight into the capacity and limits of DCS to prevent overdose and related risk behaviors.

#### Analysis of drug samples (Aim 3)

Mass spectrometry (MS) is a versatile technology that in combination with liquid (LC)- or gas (GC)-chromatographic techniques can identify virtually any chemical compound [45]. There are various MS instrument configurations, but essentially MS allows for highly accurate identification of a compound by measuring its mass to charge ratio (m/z) and analyzing its fragmentation pattern (mass spectrum) [29]. Separation of compounds is accomplished through the use of LC- or GC-columns. Importantly, LC- and GC-MS methods can distinguish between structural and/or functional analogs and can therefore identify the presence of highly potent toxic opioids such as fentanyl and its analogs within samples that contain multiple opioids [3, 46–48]. Drug samples will be transported to nearby clinical laboratories for analysis with LC- and GC-MS. Analysis results on composition (qualitative and quantitative) of drug samples will be communicated within one to two business days to frontline staff, although the ultimate goal of the pilot project is to reduce turnaround time to < 2 h; this evaluation will seek to identify process refinements that may expedite faster analysis and dissemination of results. Frontline staff will communicate results along with tailored harm reduction strategies to clients either in person or by phone.

#### Coroner and paramedic services (Aim 3)

Data-sharing agreements with the Office of the Chief Coroner for Ontario (OCC) and Toronto Paramedic Services (TPaS) will cover both de-identified individual- and aggregated-population level data on the incidence of fatal and non-fatal overdose events, including toxicology, temporal information, and geographic locations.

#### Estimated overdose incidence (Aim 3)

While no formal estimates of the overdose rate among people who use drugs in Toronto have been derived, 1002 overdose calls (fatal and non-fatal combined) were made to Toronto Paramedic Services between January 2018 and December 2018 in neighborhoods adjacent to the three frontline DCS sites, representing 31% of all calls across Toronto [49]; these neighborhoods are also those with the greatest density of people who use drugs [35]. We also note that the incidence of > 1000 overdose events recorded near DCS sites implies a relatively high event rate among potential DCS clients.

### Analytic plan

#### Multivariable model of participant overdose risk behaviors (Aim 1)

For participant survey binary outcomes, we will employ either population-averaged models (i.e., generalized estimating equations; GEE) or subject-specific models (generalized linear mixed models; GLMM) with binomial distribution and logit link. The primary outcome is recent (i.e., past 6 months) self-reported overdose; the primary exposure is a categorical measure of frequency of DCS access over the past 6 months. GEE will be used if participant visits are equally distributed over time and if we have low levels of missing data. In the case of unequal distribution of participant visits over time and/or substantial missing data, we will employ GLMM. These models efficiently address problems of missing data and variable timing of subject visits. Both GEE and GLMM are suited to the analysis of longitudinal data, as they can account for both within-subject correlation and between-subject differences with results also remaining highly interpretable. We will initially model temporal correlations between subject responses using an autoregressive structure, with subject-specific variances and correlations assumed to decline exponentially over time; this is critical given that we anticipate variance in client follow-up times. GLMM models will also include independent time-varying variables from the linked intake and post-test surveys as potential confounders. This will allow for a “pre versus post” test evaluation using longitudinal DCS data. To assess potential bias as a result of loss to follow-up, sociodemographic and drug-related factors collected on the intake and post-test surveys will be compared among participants who only completed a baseline DCS visit and those that accessed the service repeatedly. Multilevel multiple imputations may also be considered if needed.

#### Thematic analysis (Aim 2)

Thematic analysis will be employed to guide qualitative data collection and simultaneous, iterative analysis to identify themes within the data [50]. Transcripts will be reviewed multiple times by co-authors to draw on key codes, and analysis will be completed once themes reach saturation [51]. Analysis will also disaggregate responses by age, gender, racial group, sexual orientation, and drug-using behavior to identify similarities and conflicting perspectives. Respondent verification, constant comparative analysis, and multiple coding will be employed in the analysis [52, 53].

#### Longitudinal analysis of time-updated overdose data (Aim 3)

Using OCC and TPaS data, the influence of DCS on the annual incidence of fatal and non-fatal overdose events will be characterized, while adjusting for underlying trends over time. Following others [54], conditional fixed effects Poisson models will be employed to test overdose incidence counts in geographic units that do and do not have DCS present, while adjusting for the presence of SCS. This approach allows for assessment of the estimated effect of DCS by analyzing the fluctuation in incidence rates in areas before and after DCS implementation, and by then comparing the relative change in incidence to other geographic areas. Disentangling the independent effects of DCS from those of SCS presents analytic challenges. However, SCS in Toronto were operational before DCS. This stepwise implementation represents an ideal natural experiment to assess the independent effect of DCS.

#### Spatial patterns of fatal and non-fatal overdose in Toronto (Aim 3)

Employing approaches previously used to assess the association between risky drug-related behaviors and frontline services [55], data from the OCC and TPaS will be used to assess the spatial association between frontline harm reduction agencies with DCS and overdose events. This will be done by comparing geographic units that have DCS and SCS co-located with sites that only have SCS located, compared with sites that have neither. Changes over time in the incidence of overdose in these units will be determined by employing geographic information systems approaches. First, spatial buffers around each DCS site will be generated, and TPaS and OCC data will be used to capture the spatial location of non-fatal and fatal overdoses in surrounding areas. Significance testing will then be done by analyzing dissemination blocks (the smallest geographic unit defined by Statistics Canada with a population of 400–700 people). Toronto experienced 1542 non-fatal and 111 fatal overdoses over a recent 6-month period (August 2017–February 2018), and based on existing spatial data, it is estimated that the range of annual overdose events per dissemination block will range from 0 to > 100. This exploratory approach represents a simple descriptive test to determine the spatial correlation of overdoses and DCS. Second, to further account for potential confounding of the association between DCS and spatial patterns of overdose as a result of co-location of SCS and DCS [56], the mean distances between fatal overdoses (OCC data) in neighborhoods in which SCS are implemented but have not yet implemented DCS will be compared. Third, geographically weighted regression (GWR) will be employed to determine the spatial heterogeneity between the location of overdoses (dependent variable) and location of DCS (independent variable), while accounting for the presence of SCS as above. GWR calculates local spatial statistics by fitting a weighted regression equation for each dissemination block in the study area to assess local variation [57]. Local statistics will then be compared with the global statistic for the entire geographic area (i.e., the Province of Ontario), and blocks exhibiting local variation differing from the global statistic will be identified (including by undertaking a sub-analysis restricted to blocks within the City of Toronto). Using GWR, it will be determined whether those blocks exhibiting local variation in overdose incidence are more or less likely to also contain DCS. Fourth, the local annual incidence rate of overdose for each block will be determined. The Wilcoxon signed rank-test has previously been shown to be effective at assessing fluctuations in the annual rate of fatal and non-fatal overdose [56], and will allow a determination of slope differences in the overdose rate in blocks that do and do not have DCS.

### Mixed-methods analysis

A parallel mixed-methods design followed by a meta-inference process will be employed [58]. Specifically, qualitative research findings will be used to interpret survey- and laboratory-based data collection within the context of the lived experience of participants. This approach has been shown to be effective in generating an understanding of the social dimensions underpinning quantitative outcomes (e.g., use of and outcomes related to DCS, or the incidence and spatial patterns of overdose) [59–61]. Qualitative data collection and interpretation will also be used to refine the quantitative data collection process to better capture relevant factors.

### Ethics, consent, and permissions

Research Ethics Board approval has been obtained for this research study from Providence St. Joseph’s and St. Michael’s Healthcare, Toronto Public Health, Centre for Addiction and Mental Health, and University of Toronto. Client provision of informed consent is integrated into the baseline intake survey, although clients that are unwilling or unable to provide informed consent will still be provided access to DCS. Data will be securely stored at St. Michael’s Hospital.

## Results

The study addresses evidence gaps on the emerging continuum of responses to the opioid overdose crisis in Canada and elsewhere. Internationally, DCS have only targeted “recreational drug users,” and no DCS co-located with SCS and using LC- or GC-MS have been scientifically evaluated. The study therefore builds upon new and incremental lines of inquiry by evaluating the potential impact of DCS within a suite of harm reduction interventions tailored for populations at highest risk of overdose [62]. Given the rise in opioid overdose events and fatalities in Canada, the USA [63], and the UK [64], findings will provide decision makers with critical data on the potential impact of an innovative DCS model on overdose risk.

DCS in Toronto have been integrated within public health and community health agencies with existing SCS, thereby combining harm reduction service delivery with access to other services including primary care, mental health care, social service programs, and scientific evaluation. The implementation of DCS will allow frontline harm reduction agencies to extend their services by providing clients with timely information on the composition (qualitative and quantitative) of their drugs. While DCS are not a panacea for drug-related harms, increasing information on the unregulated drug supply may disrupt the risk environment for people who use drugs by equipping them with tailored and highly relevant information related to their substance use, as well as by increasing transparency and accountability in the drug market by reducing information asymmetries between buyers and sellers throughout the supply chain. Evidence from European DCS indicates that DCS benefits can extend beyond those individuals that engage directly with the service, as analysis results are often communicated to others, thus multiplying the preventive effect for a broader population of people who use drugs [65]. Although DCS can play an important role in drug market monitoring and therefore have the potential to influence drug market activities, the absence of legally regulated markets for illegal drugs creates a paradox wherein people who use drugs may become aware of dangerous adulterants in their drugs but have no viable option for the replacement of the analyzed substance. Particularly for those facing intersecting vulnerabilities such as poverty and substance-use disorders, increased information on substance composition may be insufficient to modify their risk environment. In cases where information from DCS does not effectively reduce overdose risk for clients, other policy approaches and interventions—such as scaling up low-barrier access to managed opioid programs—should be considered. Importantly, DCS have the potential to attract clients who may otherwise not access drug-related services—as has been demonstrated by DCS in Europe [18, 22, 66]—and given the co-location of DCS with other services, DCS may support increased uptake of other harm reduction, primary care, mental health care, and social services among people who use drugs. While such an integrated model of harm reduction services offers the promise of a “one-stop shop” with the potential to improve health outcomes for people who use drugs, drawbacks such as reduced anonymity may pose barriers for some clients. Accompanying evaluations of DCS and SCS will examine different models of service provision, under what conditions benefits can be maximized, suitability for specific subpopulations, and a variety of indicators including successful referrals to other services.

## Discussion

A crucial difference between the adoption of DCS in Toronto and the longstanding use of this harm reduction service throughout Europe is the targeted subpopulation of people who use drugs. Whereas most DCS globally target “recreational drug users,” in Toronto, DCS will primarily target marginalized people who use drugs accessing frontline services, many of whom use drugs regularly and by injection. This evolution in the application of DCS poses important questions that have not yet been explored, including optimal service delivery models and technologies, as well as unique barriers for this population. As the only DCS in Canada employing LC- and GC-MS analysis for point of care services, it will be important to compare results with DCS currently operating in British Columbia and Ottawa employing different and less sophisticated technologies (e.g., Fourier-transform infrared spectroscopy, fentanyl test strips), including in terms of the capability of technologies to detect drug market trends and respond to local drug use patterns. Although employing LC- and GC-MS for the analysis of drug samples has the potential to offer clients more information than the use of other technologies, the time required to receive analysis results may not be suitable for all populations of people who use drugs. It remains to be seen whether participants will consume their substance before receiving analysis results, and if they will modify their behaviors in response to analysis results, particularly when a high-potency opioid is detected.

### Limitations

The study has limitations typical of community-based intervention evaluations. Since denying access to DCS is not ethical or feasible, the recruitment process is being integrated directly into the delivery of DCS. While this will allow for the collection of data from a large proportion of the population of DCS clients, it does present limitations. For instance, it is not possible to estimate the number of individuals that will access DCS and provide data, nor the total person-time of data. Second, we note challenges in disentangling the impact of SCS and DCS on overdose incidence and related behaviors given that these two interventions will be co-located in our study setting. However, the 1-year delay between the implementation of SCS and that of DCS provides an ideal natural experiment to test the independent effects of these interventions. Further, the harmonization of data collection tools between SCS and DCS evaluations will further allow for the assessment of independent effects as we are able to evaluate outcomes among SCS clients who do and do not use DCS. Another limitation is the absence of a comparison group, except for the SCS sub-analysis. A final challenge worth noting is that while we would prefer that participants create an anonymous identifier, since this would allow us to link their data from separate visits to DCS and therefore employ panel approaches, this will depend upon the willingness of clients to consent to participating in the research, creating an anonymous identifier, and consistently recording their survey responses under their identifier at subsequent visits.

## Conclusions

Given that the impact of DCS has yet to be tested among people who use drugs at highest risk of overdose, this study will provide critical data for a range of stakeholders seeking to respond to opioid overdose crises in Canada and other settings impacted by unacceptably high rates of overdose mortality. Presenting the protocol and rationale for this study is important at this juncture, as transparency in methods will assist researchers in examining and developing DCS methods and evaluation, as well as enable the production of similar evaluations in other jurisdictions.

## Data Availability

Not applicable.

## Abbreviations

DCS: Drug checking services
GC: Gas chromatography
GEE: Generalized estimating equations
GLMM: Generalized linear mixed models
GWR: Geographically weighted regression
LC: Liquid chromatography
MS: Mass spectrometry
OCC: Office of the Chief Coroner for Ontario
SCS: Supervised consumption services
TPaS: Toronto paramedic services
